# Hypo- or conventionally fractionated radiotherapy combined with chemotherapy in patients with limited stage small cell lung cancer

**DOI:** 10.1186/s13014-017-0788-x

**Published:** 2017-03-11

**Authors:** Jing Zhang, Min Fan, Di Liu, Kuai-Le Zhao, Kai-Liang Wu, Wei-Xin Zhao, Zheng-Fei Zhu, Xiao-Long Fu

**Affiliations:** Department of Radiation Oncology, Fudan University Shanghai Cancer Center, 270 Dong an Road, Shanghai, 200032 China

**Keywords:** Limited-stage small cell lung cancer, Hypofractionated radiotherapy, Conventionally fractionated radiotherapy, Survival, Failure patterns, Toxicity

## Abstract

**Background:**

Previous data from our institution showed that hypofractionated thoracic radiotherapy (HypoTRT) with concurrent etoposide/platinum chemotherapy yielded favorable survival in patients with limited-stage small cell lung cancer (LS-SCLC). The present study retrospectively compared the survival outcomes, failure patterns and toxicities between groups of LS-SCLC patients treated with conventionally fractionated thoracic radiotherapy (ConvTRT) or HypoTRT combined with chemotherapy.

**Methods:**

Medical records of LS-SCLC patients between January 2010 and December 2013 at Fudan University Shanghai Cancer Center were retrospectively reviewed. All patients treated with chemotherapy and ConvTRT (2 Gy per fraction daily, DT ≥ 56 Gy) or HypoTRT (2.5 Gy per fraction daily, DT = 55 Gy) were eligible for analysis. Progression-free survival (PFS) and overall survival (OS) were generated for different populations using the Kaplan-Meier method and compared using the log-rank test. Comparisons of failure patterns and toxicity were analyzed using the *χ* ^2^ test.

**Results:**

A total of 170 patients treated with HypoTRT (*n* = 69) or ConvTRT (*n* = 101) were eligible for analysis. The median PFS and OS were 13.7 and 25.3 months, respectively, in the ConvTRT cohort, which was similar to the HypoTRT cohort (PFS 18.2 months, *p* = 0.991, and OS 27.2 months, *p* = 0.698), with a median follow-up of 30 months. Multivariate analysis revealed that PCI and TNM stage were prognostic factors for PFS and that PCI was prognostic for OS. The patterns of failure (stratified by local-regional recurrence, distant metastasis or both as first relapse) were similar between the dose cohorts (*p* = 0.693, *p* = 0.330, *p* = 0.572). Distant metastasis remained the main failure pattern. The brain was the most frequent remote failure site, followed by bone, liver and adrenal gland. PCI improved the 2-year survival rate from 46.1% to 70.0% and the 2-year PFS rate from 20.9% to 45.3%, respectively (*p* < 0.001). Grade ≥3 esophagitis and pneumonitis occurred in 9.9% and 11.9%, respectively, of the patients in the ConvTRT cohort and in 11.6% and 10.0%, respectively, of those in the HypoTRT cohort (*p* = 0.815).

**Conclusion:**

This retrospective analysis demonstrated that HypoTRT or ConvTRT combined with etoposide/platinum chemotherapy yielded statistically similar survival, treatment failure outcomes, and toxicity profiles. PCI correlated with improved PFS and OS.

## Background

Lung cancer remains the leading cause of cancer-related deaths worldwide [1]. Small-cell lung cancer (SCLC) accounts for almost 13% of all new cases, and it is distinguished from non-small-cell lung cancer (NSCLC) by a more rapid doubling time, higher growth fraction and early metastatic dissemination [2]. Limited stage small cell lung cancer (LS-SCLC) is diagnosed in approximately one-third of patients, and it is potentially curable. The combined-modality treatment of chemotherapy and thoracic radiotherapy (TRT), particularly concurrent chemoradiotherapy (CRT) for those with good performance status, followed by prophylactic cranial irradiation (PCI) is the standard of care for LS-SCLC [3]. The addition of TRT to systemic chemotherapy improves long-term survival and local control [4, 5]. Nearly 20% of the LS-SCLC patients who received concurrent CRT achieved 5-year survival with a median overall survival (OS) of 19.9 months [6].

A current consensus of the optimal TRT dose fraction regimen is lacking. The hallmark INT0096 study favored twice-daily 45 Gy in 3 weeks (1.5 Gy per fraction) with concurrent cisplatin/etoposide chemotherapy, which significantly improved the OS compared to once-daily 45 Gy in 5 weeks (1.8 Gy per fraction) [7]. Hyperfractionated-accelerated radiotherapy became one of the recommended standard TRT regimens. However, this study was continuously debatable for the low biological equivalent dose (BED) in the once-daily group and the high rate of grade 3 or worse esophagitis in the twice-daily group. The Cancer and Leukemia Group B (CALGB) conducted a series of studies in which conventionally fractionated thoracic radiotherapy (ConvTRT) with high dose of 70 Gy was applied concurrently with chemotherapy to achieve improvements in survival and local control with tolerable radiation-associated toxicities [6, 8–11]. The phase III study (CONVERT) compared concurrent twice-daily CRT (45 Gy/30 F) with once-daily CRT (66 Gy/33 F) in patients with LS-SCLC and showed similar survival outcomes and toxicities, with the exception of significantly more grade 3/4 neutropenia in the twice-daily arm. The CONVERT results supported the use of either once-daily or twice-daily RT as the standard of care for patients with LS-SCLC [12].

Nevertheless, twice-daily RT was not widely adopted because of toxicity and inconvenience. Once-daily RT remained the most commonly used regimen for LS-SCLC. Once-daily HypoTRT with a shortening of the overall treatment time may optimize survival and tumor control [13]. Murray et al. [14] reported a favorable efficacy of HypoTRT (40 Gy/15 F) early in 1993, during the era of two-dimensional radiotherapy, with a median OS and 5-year survival rate of 21.2 months and 22%, respectively. An ensuing phase II study of HypoTRT 55 Gy (2.5 Gy per fraction) and concurrent chemotherapy achieved a median OS of 28.5 months and a 2-year survival rate of 58.2% [15]. LS-SCLC patients in our institution were treated with once-daily TRT (either ConvTRT or HypoTRT) combined with chemotherapy. Few studies have compared the ConvTRT and HypoTRT. The present study retrospectively compared the survival outcomes, failure patterns and toxicities between ConvTRT and HypoTRT in combination with etoposide/platinum chemotherapy in LS-SCLC patients between January 2010 and December 2013.

## Methods

### Patients

A database of patients with histologically or cytologically proven SCLC at Fudan University Shanghai Cancer Center between January 2010 and December 2013 was retrospectively reviewed. Routine staging evaluation of SCLC consisted of a medical history and physical examination, CBC and comprehensive chemistry panel with renal and hepatic function tests, contrast-enhanced chest CT scans, contrast-enhanced abdomen CT scans or abdomen ultrasound, bone scan, and MRI or CT scan of the brain. Whole-body PET-CT was not mandatory. The initial staging of LS-SCLC was based on the Veterans Administration Lung Study Group (VALSG) two-stage classification and a modified staging system proposed by the International Association for the Study of Lung Cancer (IASLC) [16]. Except for contralateral hilar lymph node involvement and ipsilateral pleural effusions, patients with contralateral mediastinal or bilateral supraclavicular lymph node metastases were eligible for analysis.

### Treatment delivery

Patients were immobilized in the supine position with a thermoplastic head and shoulder mask or on a vacuum pad with arms placed over the head. A simulation CT with slice thickness of 5 mm was performed. CT images were imported to a treatment planning system to create a treatment design. All patients received induction chemotherapy. The gross tumor volume (GTV) contouring of primary tumor in lung, mediastinal lymph nodes and involved lymph nodal regions were based on pretreatment chest CT and/or PET scan, and restage chest CT. The clinical target volume (CTV) was not outlined in these patients. The planning target volume (PTV) was generated with a margin of 1.0-1.5 cm added to GTV.

All patients were treated using the 3D-CRT or IMRT technique. The volume of PTV that received less than 95% of the prescription dose should not exceed 1%, and the volume of PTV that received less than 99% of the prescription dose should not exceed 5%. The dose tolerance of organs at risk was constrained. The following parameters were used for the HypoTRT cohort: maximum spinal cord dose ≤42 Gy; V_20_ (percentage of lung volume receiving > 20 Gy) ≤ 25%; mean lung dose ≤14.5 Gy; and mean heart dose ≤27 Gy. The following parameters were used for the ConvTRT cohort: maximum spinal cord dose ≤45 Gy; V_20_ (percentage of lung volume receiving > 20 Gy) ≤ 30%; mean lung dose ≤15 Gy; and mean heart dose ≤30 Gy.

The chemotherapy regimens were EP or EC. The EP regimen consisted of cisplatin (25 mg/m^2^/days 1–3) and etoposide (100 mg/m^2^/days 1–3). The EC regimen consisted of carboplatin (AUC 5-6/day 1) and etoposide (100 mg/m^2^/days 1–3). Chemotherapy was administered every 3–4 weeks for four to six cycles, and the doses were modified according to the toxicity grade by the treating physicians. Tumor response was assessed using Response Evaluation Criteria in Solid Tumors (RECIST) version 1.0. Toxicity was assessed using Common Terminology Criteria Adverse Events (CTCAE) version 3.0 of the National Cancer Institute (NCI). All patients underwent CBC, comprehensive chemistry panel with renal and hepatic function tests, and CT of the chest with intravenous contrast at 1 month after the completion of therapy. Patients who achieved partial or complete tumor response in the chest were considered for PCI. The follow-up times were typically 3 months for the first year and 6 months for the next 2 years. Chest CT and abdomen CT or ultrasound examination were routinely performed during follow-up. Brain MRI or CT and bone scan were performed when symptoms were reported.

### Statistical analysis

The Statistical Package for Social Sciences (SPSS version 20.0, IBM, USA) software was used for statistical analyses. Comparisons of categorical variables were performed using Fisher’s exact test or the *χ* ^2^ test. Independent-samples *T* test was used for continuous measures to assess the differences between the groups. The Kaplan-Meier method was used to estimate overall survival (OS), progression-free survival (PFS), time to locoregional recurrence, and distant metastasis. OS was defined as time from the initiation of treatment to death. PFS was defined as time from the initiation of treatment to disease progression or death. Time to locoregional recurrence was defined as time from the initiation of treatment until the date of locoregional failure as a first event. Time to distant metastasis was similarly defined. Log-rank tests were used to evaluate differences in the time-to-event outcomes between the groups with two-sided *p* values. Statistical significance was defined as a p-value less than 0.05. We used the Cox regression model to detect whether associated clinical factors were predictors for OS and PFS.

## Results

### Patient characteristics

A total of 170 patients treated with ConvTRT (2 Gy per fraction daily, median 60 Gy/30 F, range from 56 Gy/28 F to 66 Gy/33 F**)** or HypoTRT (2.5 Gy per fraction daily, DT = 55 Gy) combined with etoposide/platinum chemotherapy were eligible for analysis. Nine patients who received a hyperfractionated regimen were excluded from the analysis. Sixty-nine of the 170 patients received HypoTRT, and 101 patients received ConvTRT. Table 1 summarizes the baseline characteristics of the patients. Patient- and treatment-related variables were comparative between the two cohorts, except for PCI and CRT modality. Sixty-three patients (91.3%) received concurrent CRT in the HypoTRT group and 53 patients started early concurrent HypoTRT with the 1st to 3rd cycle of chemotherapy. Fifty patients (49.5%) were treated with concurrent CRT in the ConvTRT group and 34 patients received early concurrent ConvTRT (*p* < 0.001). PCI (25 Gy/10 F) was administered to 66.7% and 47.5% of the patients with partial or complete response in chest tumors in the HypoTRT and ConvTRT cohorts (*p* = 0.014), respectively.

**Table 1:** Patient demographics and clinical characteristics

Characteristics	HypoTRT group (*n* = 69)	ConvTRT group (*n* = 101)	*P*
Gender			0.414
Male	57(82.6%)	88(87.1%)	
Female	12(17.4%)	13(12.9%)	
Age (yr)			0.698
Median	59	57	
Range	31–76	38–83	
Smoking			0.403
Yes	48(69.6%)	64(63.4%)	
No	21(30.4%)	37(36.6%)	
Performance status(ECOG)			0.564
0–1	65(94.2%)	92(91.1%)	
2	4(5.8%)	9(8.9%)	
TNM stage			0.761
I-II	10(14.5%)	13(12.9%)	
III	59(85.5%)	88(87.1%)	
CRT modality			<0.001
Concurrent CRT	63(91.3%)	50(49.5%)	
Early radiotherapy	53	34	
Late radiotherapy	10	16	
Sequential CRT	6(8.7%)	51(50.5%)	
Cycles of chemotherapy			0.924
<4 cycles	12(17.4%)	17(16.8%)	
≥4 cycles	57(82.6%)	84(83.2%)	
PCI			0.014
Yes	46(66.7%)	48(47.5%)	
No	23(33.3%)	53(52.5%)	

*Abbreviations: PCI* prophylactic cranial irradiation

*CRT* chemoradiotherapy

*ECOG* Eastern Cooperative Oncology Group

### Response evaluation

Patients were assessed using chest CT scans according to the RECIST version 1.0 after the completion of CRT. The overall objective response rate was 97%, with complete and partial response achieved in 32% and 65% of the patients, respectively, and 2 patients (3%) achieved stable disease in the HypoTRT group. Twenty-eight patients in the ConvTRT group exhibited complete response (28%), 69 patients exhibited partial response (68%), and 4 patients exhibited stable disease (4%). There was no significant difference in the response rate between the groups (*p* = 0.806).

### Survival

**Fig. 1:**
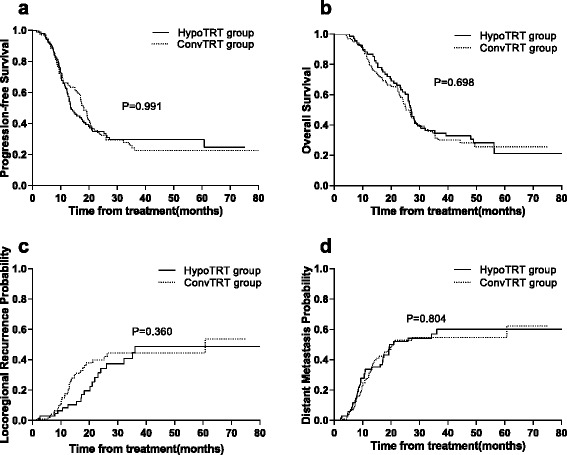
**a** Progression-free survival for patients in the HypoTRT and ConvTRT groups. **b** Overall survival for patients in the HypoTRT and ConvTRT groups. **c** Locoregional recurrence probability for patients in the HypoTRT and ConvTRT groups. **d** Distant metastasis probability for patients in the HypoTRT and ConvTRT groups

The last follow-up date was November 2016. Nine patients were lost to follow-up. The median follow-up time was 30 months, with a range from 4 to 82 months. Of the 170 patients, 120 (70.6%) patients suffered treatment failure: 52 in the HypoTRT group and 68 in the ConvTRT group. The median PFS was 17.0 months (95%CI: 14.0–20.0) for the entire group of patients. The median PFS was 18.2 months (95% CI: 15.8–20.6) and 13.7 months (95% CI: 11.3–16.1) for the HypoTRT and ConvTRT cohorts, respectively (*p* = 0.991). The 1-year and 2-year PFS rates were 64.8% and 32.4%, respectively, for patients who received HypoTRT compared to 61.6% and 33.5%, respectively, for patients who received ConvTRT (Fig. 1a).

One hundred and nineteen of the 170 patients (70%) died: 49 patients in the HypoTRT group and 70 patients in the ConvTRT group. The median OS was 26.8 months (95% CI: 24.9–28.7) for all patients, 27.2 months (95% CI: 25.2–29.2) for patients who received HypoTRT, and 25.3 months (95% CI: 22.5–28.1) for patients who received ConvTRT (*p* = 0.698). The 1- and 2-year survival rates were 87.0% and 62.2%, respectively, for patients in the HypoTRT group compared with 82.2% and 57.3%, respectively, of the patients treated with ConvTRT (Fig. 1b).

### Patterns of failure

Table 2 shows the failure patterns for all patients. Twelve patients in the HypoTRT group (17.4%) had locoregional failure, 29 (42.0%) patients developed distant metastasis, and 11(15.9%) patients exhibited both failure patterns as first recurrence. Twenty (19.8%) of the patients who received ConvTRT had locoregional failure, 35 (34.7%) patients exhibited distant metastasis, and 13 (12.9%) patients exhibited both failure patterns as first recurrence. The locoregional failure included in-field failure and recurrence outside the PTV but within the ipsilateral hilar, bilateral mediastinal or bilateral supraclavicular nodal basin region. The 1-year locoregional failure rates were 10.3% versus 17.6%, and the 2-year locoregional failure rates were 34.3% versus 42.1% for the HypoTRT and ConvTRT arms, respectively (*p* = 0.360) (Fig. 1c).

**Table 2:** Failure patterns in the HypoTRT and ConvTRT groups

	HypoTRT group (*n* = 69)	ConvTRT group (*n* = 101)	*P*
Failure rates	52(75.4%)	68(67.3%)	0.259
Initial failure pattern			
Locoregional failure	12(17.4%)	20(19.8%)	0.693
In field	6	9	
Out of field	3	4	
Both	3	7	
Distant	29(42.0%)	35(34.7%)	0.330
Liver only	4	3	
Bone only	5	5	
Brain only	7	13	
Adrenal gland only	2	1	
Multiple sites	9	8	
Other sites	2	5	
Both locoregional and distant failure	11(15.9%)	13(12.9%)	0.572

Distant metastasis was the main failure pattern for this group of patients. The 1- and 2-year distant metastasis rates were 35.2% and 54.2%, respectively, for the patients treated with HypoTRT and were 29.4% and 52.8%, respectively, for patients treated with ConvTRT. No statistically significant differences in distant metastasis were observed between groups (*p* = 0.804) (Fig. 1d). The most common site of distant metastasis was the brain (20), followed by bone (10), liver (7) and adrenal gland (3). Brain metastases were diagnosed to the last follow-up in 10.6% of the patients (10/94) who received PCI and in 35.5% (27/76) of the patients who did not receive PCI (*p* < 0.001). The median OS and PFS were 32.0 and 20.1 months, respectively, in patients who received PCI compared to 23.0 and 12.3 months, respectively, in patients who did not receive PCI (*p* < 0.001). PCI reduced the 2-year mortality rate from 53.9% to 30.0% and the 2-year disease-progression rate from 79.1% to 54.7%.

### Toxicity

Table 3 lists acute toxicities by site and grade. The most common radiation-associated acute complication was esophagitis, which was experienced by 11.6% and 9.9% of the patients in the HypoTRT and ConvTRT arms, respectively. One patient in the HypoTRT group and 2 patients in the ConvTRT group died of treatment-related pneumonitis. Grade 3 to 4 leukopenia, granulocytopenia, thrombocytopenia and anemia occurred in 55.1% and 40.6%, 52.2% and 42.6%, 28.9% and 20.8%, 10.1% and 14.8% of HypoTRT or ConvTRT patients, respectively. No significant differences in acute toxicities were demonstrated between the TRT cohorts.

**Table 3:** Acute toxicities in the HypoTRT and ConvTRT groups

Acute toxicities	HypoTRT group (*n* = 69)			ConvTRT group (*n* = 101)			*P*
Grade	3	4	5	3	4	5	
Esophagitis	8(11.6%)	0	0	10(9.9%)	0	0	0.725
Pneumonitis	5(7.2%)	1(1.4%)	1(1.4%)	8(7.9%)	2(2.0%)	2(2.0%)	0.639
Leukopenia	30(43.5%)	8(11.6%)	0	29(28.7%)	12(11.9%)	0	0.063
Granulocytopenia	26(37.7%)	10(14.5%)	0	32(31.7%)	11(10.9%)	0	0.218
Thrombocytopenia	13(18.8%)	7(10.1%)	0	15(14.9%)	6(5.9%)	0	0.220
Anemia	6(8.7%)	1(1.4%)	0	9(8.9%)	6(5.9%)	0	0.369

### Prognostic factors

Univariate analysis indicated that various potential prognostic factors including gender, TNM stage, CRT modality (concurrent vs. sequential), cycles of chemotherapy, and PCI may influence survival. Early TRT defined as beginning with 1–3 cycle of chemotherapy was not prognostic for either PFS or OS. Multivariate analysis revealed that PCI was prognostic factor for OS (*p* = 0.003, HR = 0.528, 95%CI: 0.348–0.803) and that TNM stage and PCI were prognostic factors for PFS (*p* = 0.016, HR = 2.313, 95%CI: 1.169–4.578; *p* = 0.001, HR = 0.512, 95%CI: 0.340–0.772).

## Discussion

SCLC presented high sensitivity to CRT. The integration of TRT and etoposide/platinum chemotherapy made LS-SCLC potentially curable [7]. A meta-analysis [5] in the early 1990s reported that the addition of TRT to systemic chemotherapy resulted in a 14% reduction in mortality rate and 5.4% increase in 3-year survival. Another meta-analysis [4] found that TRT improved 2-year survival by 5.4% and intrathoracic tumor control by 25.3%. RT schedules (Hypo-, Conv-, and HyperTRT) for LS-SCLC have been extendedly studied over the past several decades. Hyperfractionated-accelerated radiation of 45 Gy in 1.5 Gy twice-daily fractions with concurrent cisplatin plus etoposide chemotherapy is a standard TRT regimen that yielded more favorable outcomes with a median survival of 23.0 months and 5-year OS rate of 26.0% compared to once-daily radiation of 45 Gy (1.8 Gy per fraction) in the INT0096 study [7]. However, this radiation regimen failed to gain widespread acceptance because of the inconvenience and high rate of severe esophagitis (32.0%) [7]. Once-daily ConvTRT is still the most commonly used regimen in clinical practice.

The HypoTRT is increasingly adopted in current strategy to treat early and/or locally advanced NSCLC. Nevertheless, the efficacy of HypoTRT in patients with LS-SCLC remained unsolved. In a phase II study from our institution, Xia et al. [15] reported a favorable median 2-year progression free survival of 49% and overall survival of 58.2% in 59 LS-SCLC patients treated with concurrent HypoTRT (55 Gy/22 F) and etoposide/cisplatin. However, earlier data from Cleveland Clinic [17] revealed that changing from a HypoTRT (40 Gy/15 F) to a ConvTRT (50 Gy/25 F) prescription did not alter survival or toxicity outcomes. Herein, we retrospectively compared two radiation regimens (ConvTRT 56–66 Gy, 2.0 Gy per faction daily and HypoTRT 55 Gy, 2.5 Gy per faction daily) combined with etoposide/platinum chemotherapy in 170 consecutive patients with LS-SCLC.

Even though most patient- and treatment-related variables including response rate were comparable, PCI rate and CRT modality were not balanced between the two cohorts. There were significantly more patients in the HypoTRT cohort receiving early concurrent CRT and PCI. Nevertheless, no statistically significant differences were found between cohorts in OS or PFS. The cumulative incidences of 2-year survival and PFS were 57.3% vs. 62.2% (*p* = 0.698) and 33.5% vs. 32.4% (*p* = 0.991), respectively, for the ConvTRT and HypoTRT cohorts. The survival data of HypoTRT cohort was close to those reported by Xia et al. in the phase II trial [15]. In univariate analysis, early TRT defined as beginning with 1–3 cycle of chemotherapy was not prognostic factor for either PFS or OS. This was consistent with the systemic review from Fried et al. [18] and the meta-analysis from Spiro et al. [19], which suggesting that the improvement from early TRT was small and often not evident for once-daily fractionation.

In our study, multivariate analysis revealed that PCI was a prognostic factor for both OS and PFS. PCI is an effective treatment to prevent brain metastasis in LS- or ES-SCLC. A meta-analysis in 1999 demonstrated that PCI in LS-SCLC patients with CR resulted in an absolute decrease of 25.3% in the cumulative incidence of brain metastasis at 3 years from 58.6% to 33.3% and an absolute improvement in survival at 3 years from 15.3% to 20.7% [20]. However, only a portion of patients with response received PCI in clinical practice. Patel et al. [21] reported that PCI was only delivered in 8% patients by reviewing LS-SCLC data between 1988 and 1997 in the SEER program and that the 5-year survival rate was 19% in patients who received PCI compared to 11% in patients who did not receive PCI (*p* < 0.01). PCI was administered in 55% of the LS-SCLC patients in our study. PCI improved the 2-year survival rate from 46.1% to 70.0% and the 2-year PFS rate from 20.9% to 45.3%, respectively (*p* < 0.001). In this group of patients, PCI reduced brain metastasis rate from 35.5% (27/76) to 10.6% (10/94), and was correlated with improved PFS and OS, highlighting the importance of PCI for those responders. Nevertheless, this finding should be interpreted with caution, because a higher proportion of patients receiving PCI were treated with concurrent CRT and more cycles of chemotherapy. The effect of multiple testing cannot be ruled out.

The once-daily HypoTRT regimen became more practicable in recent years because of improved normal tissue protection via 3DCRT and IMRT. A shortened duration of the HypoTRT regimen may decrease cancer cell repopulation [13]. Locoregional recurrence in the present study occurred in 32.9% of patients, and there was no difference between the HypoTRT (33.3%) and ConvTRT (32.7%) cohorts. Distant failure often precedes locoregional recurrence and presents as the primary pattern of failure because of the early dissemination behavior of SCLC. In our report, 120 (70.6%) patients experienced disease progression, which was most frequently distant metastasis (51.8%). Forty patients (58.0%) in the HypoTRT group and 48 (47.5%) patients in the ConvTRT group with or without locoregional recurrence experienced initial distant metastasis at the last follow-up.

Few significant acute toxicities were observed in the current study. The most common Grade 3 or worse acute toxicity was myelosuppression. A total of 11.6% and 11.9% of patients in the HypoTRT and ConvTRT group, respectively, experienced grade 4 leukopenia. The main radiation-associated toxicities were esophagitis and pneumonitis. Severe esophagitis was reported in 9.9% of patients in the ConvTRT cohort and 11.6% of patients in the HypoTRT cohort in our study. The rate of pneumonitis was 11.9% in the ConvTRT arm and 10.0% in the HypoTRT arm. Watkins et al. [22] reported that severe esophagitis and pneumonitis with once-daily TRT occurred in 24.0% and 6.0% of patients, respectively, compared with 20.0% and 4.0% in the twice-daily group, and one patient treated with twice-daily TRT died of treatment-associated pneumonitis. However, in a recent phase III trial [23], grade 3–4 esophagitis was 0.9%–3.6% for Korean patients who received concurrent CRT. Overall, the toxicities in our study seemed tolerable and acceptable. Notably, all patients enrolled in our study received 3D-CRT or IMRT treatment on the basis of involved-field radiotherapy.

There are certain limitations of our study. It was retrospective and the sample size was not large enough. The PCI rate and timing of TRT were not balanced between the two arms of patients who received Conv- or HypoTRT. A higher proportion of patients received early TRT and PCI in the HypoTRT arm. The selection bias may affect the results of the statistical analysis.

## Conclusions

This retrospective analysis demonstrated that HypoTRT or ConvTRT combined with etoposide/platinum chemotherapy yielded statistically similar survival, treatment failure outcomes, and toxicity profiles. PCI correlated with improved PFS and OS.
